# In Vitro Induction of Pluripotency from Equine Fibroblasts in 20% or 5% Oxygen

**DOI:** 10.1155/2020/8814989

**Published:** 2020-11-26

**Authors:** Raquel V. G. de Castro, Naira C. G. Pieri, Paulo Fantinato Neto, Bianca M. Grizendi, Renata G. S. Dória, Flavio V. Meirelles, Lawrence C. Smith, Joaquim M. Garcia, Fabiana F. Bressan

**Affiliations:** ^1^Department of Pathology, Reproduction and One Health, Faculty of Agricultural and Veterinary Sciences, São Paulo State University, 14884-900, Brazil; ^2^Department of Veterinary Medicine, Faculty of Animal Science and Food Engineering, University of São Paulo (FZEA/USP), 13635-900, Brazil; ^3^Department of Animal Reproduction, School of Veterinary Medicine and Animal Science, University of São Paulo (FMVZ/USP), 13635-900, Brazil; ^4^Centre de Recherche en Reproduction et Fertilité, Faculté de Médecine Vétérinaire, Université de Montréal, QC, Canada J2S 2M2

## Abstract

The cellular reprogramming into pluripotency is influenced by external and internal cellular factors, such as *in vitro* culture conditions (e.g., environmental oxygen concentration), and the aging process. Herein, we aimed to generate and maintain equine iPSCs (eiPSCs) derived from fibroblasts of a horse older than 20 years and to evaluate the effect of different levels of oxygen tension (atmospheric 20% O_2_, 5% O_2_, or 20% to 5% O_2_) on these cells. Fibroblasts were reprogrammed, and putative eiPSCs were positive for positive alkaline phosphatase detection; they were positive for pluripotency-related genes *OCT4*, *REX1*, and *NANOG*; immunofluorescence-positive staining was presented for OCT4 and NANOG (all groups), SOX2 (groups 5% O_2_ and 20% to 5% O_2_), and TRA-1-60, TRA-1-81, and SSEA-1 (only in 20% O_2_); they formed embryoid bodies; and there is spontaneous differentiation in mesoderm, endoderm, and ectoderm embryonic germ layers. In addition to the differences in immunofluorescence analysis results, the eiPSC colonies generated at 20% O_2_ presented a more compact morphology with a well-defined border than cells cultured in 5% O_2_ and 20% to 5% O_2_. Significant differences were also observed in the expression of genes related to glucose metabolism, mitochondrial fission, and hypoxia (*GAPDH*, *GLUT3*, *MFN1*, *HIF1α*, and *HIF2α*), after reprogramming. Our results show that the derivation of eiPSCs was not impaired by aging. Additionally, this study is the first to compare high and low oxygen cultures of eiPSCs, showing the generation of pluripotent cells with different profiles. Under the tested conditions, the lower oxygen tension did not favor the pluripotency of eiPSCs. This study shows that the impact of oxygen atmosphere has to be considered when culturing eiPSCs, as this condition influences the pluripotency characteristics.

## 1. Introduction

Considering the importance of therapeutic alternatives for studying and treating diseases, induced pluripotent stem cells (iPSCs) appear to be a promising alternative. In contrast to cultured primary cardiomyocytes and neuronal cells that show limited proliferation *in vitro*, are obtained through invasive procedures, and are usually not from the patient who will receive them for treatment, iPSCs are unlimited sources of all types of cells, have a high differentiation capacity, and can be reprogrammed from any type of cell and maintained in culture for long periods [1, 2].

Studies on stem cells and regenerative cell therapies in companion animals have largely contributed to advances in basic biology and clinical applications in humans [3]. For example, equines are frequently exposed to intense physical activity and frequently present with musculoskeletal injuries, such as bone fractures, muscular injuries, and osteoarthritis-effected tendons, making them ideal models to study correlated human diseases, allowing for testing of new treatments and drugs, and translating regenerative techniques to humans [4–6].

Furthermore, equine is an interesting species for studying aging, as these animals usually get older. With aging, significative diseases and injuries appear in these animals, as cardiac and gastrointestinal tract disease, musculoskeletal problems, respiratory tract problems, and skin and ophthalmologic disorders, among others [7–10]. Important changes in cell metabolism and energy production and utilization occur causing telomere erosion, genome damage, and mitochondrial dysfunction at the cellular level [11, 12]. Thus, studies of aged equines are important to improve the quality of life and longevity of these animals.

However, in regard to using iPSCs for the treatment of disorders that emerge with aging, it is important to highlight that factors such as cellular senescence, advanced age of the cell donor, and a high number of passages of cells *in vitro* are detrimental to the reprogramming of human and murine fibroblasts [13–15], reinforcing the need for strategies to circumvent these limitations. One possible method for improving reprogramming efficiency involves controlling the oxygen tension in the cell culture. A few studies on human and mouse cells have already reported an improved generation of iPSCs under conditions of lower oxygen tension [14, 16–19].

This strategy relies on the fact that iPSCs and ESCs, which are highly proliferative cells, have a different energetic metabolism compared to differentiated quiescent cells, as they preferentially use the glycolytic pathway for producing energy instead of oxidative phosphorylation (OXPHOS) [20, 21], and inducing this metabolic shift improves reprogramming. This preference is explained by the fact that glycolysis generates ATP more rapidly than does OXPHOS, and furthermore, glucose degradation provides important building blocks for the generation of important cellular components, such as nucleotides, amino acids, and lipids, which supply the needs of a highly proliferating cell [21–23].

In equine species, relatively few studies have reported iPSC acquisition, and none of them considered the effects of oxygen tension. Thus, studies in an oxygen-controlled atmosphere are needed to elucidate the influence of the atmospheric oxygen level on equine species. In addition, no attempts have been made to generate eiPSCs from considerably older equines.

Herein, we aimed to reprogram skin fibroblasts from a horse older than 20 years and studied the possible effects of oxygen tension based on high oxygen levels (group H—20%), reduced oxygen from high to low levels (group HL—from 20% to 5%), and low oxygen (group L—5%), during reprogramming *in vitro* culture. The results showed successful reprogramming of fibroblasts cultured with either a high or low oxygen concentration. eiPSCs were positive for alkaline phosphatase, expressed endogenous pluripotency-related genes, and were positive for pluripotency markers, according to immunofluorescence tests, and were able to form embryoid bodies positive for endoderm, mesoderm, and ectoderm markers. Considerable differences were found between the groups, as group H presented with colonies of distinct morphology and a unique immunofluorescence marker profile. Moreover, differences in senescence-, glucose metabolism-, mitochondrial fusion-, and hypoxia-related genes were found. These results show that oxygen influences the reprogramming process, generating pluripotent cells with different profiles.

## 2. Materials and Methods

All procedures were submitted to and approved by the Ethics Committee on Animal Use (CEUA) of the School of Animal Science and Food Engineering, São Paulo University (FZEA/USP, protocol number 5153150218).

### 2.1. Adult Fibroblast Isolation and Culture

For fibroblast acquisition, a skin fragment was collected from the dorsal lateral metacarpophalangeal region of a male horse more than 20 years old and three young males (3 to 5 years old). All animals were healthy and located at the Veterinary Medicine Department (FZEA/USP). The skin fragments were taken to the lab, and fibroblasts were recovered after 3 hours of digestion with collagenase IV (1 mg/ml, #C2674, Sigma-Aldrich). The fibroblasts were then separated and seeded in 5% CO_2_ in atmospheric air (fibroblasts under high O_2_ tension or FH) or 5%O_2_ + 5%CO_2_ + 90%N_2_ (fibroblasts under low O_2_ tension or FL), both at 38.5°C. To guarantee the ideal oxygen concentration for the FL, a HERA Cell VIOS 160i (Thermo Scientific) incubator was used and the conditions were analyzed weekly with a Bacharach combustion test kit with Fyrite 0-21% and 0-60% O_2_ indicator fluid. The fibroblasts were cultured in IMDM (#12200036, Thermo Fisher) with 10% fetal bovine serum (#SH30071.03, HyClone), 0.1 mM nonessential amino acids (#11140050, Thermo Fisher), and 1% penicillin/streptomycin (#15070063, Thermo Fisher). Before reaching 90% confluence, the cells were passaged and frozen.

### 2.2. Population Doubling Time

For the determination of population doubling time (PDT), the FH and FL of three different young horses (3 to 5 years old) and those of a horse older than 20 years were all plated in triplicate in 6-well plates at a concentration of 3 × 10^4^ cells per well and kept in culture at the respective oxygen tensions for 48 hours. They were then resuspended with TrypLE Express (#12604021, Thermo Fisher Scientific), counted, and replated at 3 × 10^4^ cells per well. This procedure was repeated for 5 passages. To calculate the PDT, the following equation was applied, according to Gruber et al. (2012): PDT = (*T* − *T*0) log 2/(log *N* − log *N*0), where PDT is population doubling time, (*T* − *T*0) is time between the counting (hours), *N*0 is the number of plated cells at the beginning, and *N* is the number of counted cells. For statistical analysis, the data were analyzed in SAS University Edition software, and a Shapiro-Wilk test was performed to test for a normal distribution. The results were subjected to analysis of variance followed by the Bonferroni test to compare different experimental groups. The significance level was 1% for all analyses.

### 2.3. Induced Cellular Reprogramming

*In vitro* cellular reprogramming was performed as described by Pessôa et al. and Bressan et al., with minor alterations [24, 25], using the fibroblasts from an aged equine. Briefly, a polycistronic lentiviral vector (STEMCCA, Millipore) containing the human sequences for *OCT4*, *SOX2*, *KLF4*, and *c-MYC* (hOSKM) was used to transduce FH and FL from an aged equine. Lentiviral vector production was performed by lipofection (Lipofectamine 3000, #L3000015, Life Technologies) of 6 × 10^6^ 293FT cells with 12 *μ*g of hOSKM vector; 1.2 *μ*g of auxiliary TAT, REV, and Hgpm2 vectors; and 2.4 *μ*g of VSVG for 6 hours. The culture medium was collected after 24, 48, and 72 hours, filtered, ultracentrifuged at 48960 g for 1 hour and 40 minutes, and used for transduction.

FH and FL were seeded in 6-well plates (2 × 10^4^ cells per well), always respecting the original oxygen tension levels during the process. The hOSKM lentivirus vector was used for overnight transduction in the presence of 8 *μ*g/ml polybrene (hexadimethrine bromide, #H9268, Sigma-Aldrich). Supplementation with 0.5 mM sodium butyrate (#B5887, Sigma-Aldrich) was performed from day 3 to day 12. After six days, the cells were recovered and plated (4.75 × 10^4^ cells per well) onto feeder layers of mouse embryonic fibroblasts (MEFs) treated with mitomycin C (#M4287, Sigma-Aldrich). A third group was created in order to study the effects of oxygen tension alteration during reprogramming and culture: one-half of the cells from group H (20% O_2_) were passaged in low oxygen (5%), creating a high-to-low (HL) group. Thus, the H and HL groups originated from group FH, and the L group originated from group FL.

On day six, the culture medium was replaced by eiPSC media consisting of DMEM/F12 KO (#12660-012, Thermo Fisher) with 20% *knockout serum replacement* (#10828010, Thermo Fisher), 2 mM GlutaMAX (#35050061, Thermo Fisher), 0.1 mM of MEM nonessential amino acids (#11140050, Thermo Fisher) with 0.1 mM 2-mercaptoethanol (#M6250, Sigma-Aldrich), 1% penicillin/streptomycin (#15070063, Thermo Fisher), and 10 ng/ml bFGF (#100-18B, PeproTech).

### 2.4. Characterization of Equine iPSCs (eiPSCs)

The cells were observed daily until the first colonies appeared. The eiPSCs were first recognized by their morphology, and once the eiPSC colonies were formed, the reprogramming efficiency was calculated by dividing the number of colonies by the number of seeded cells. Alkaline phosphatase (AP) detection was performed with a *leukocyte alkaline phosphatase kit* (#86R, Sigma-Aldrich). Three AP-positive colonies from each group were maintained in culture and collected for qRT-PCR at an early passage (EP—passage 5) and late passage (LP—passage 16). One colony from each group was selected for the immunocytochemistry assay at passages 16 and 30 and for an embryoid body formation assay at passage 30.

### 2.5. Immunofluorescence for Determining Pluripotency and the Spontaneous Differentiation of Embryoid Bodies

The eiPSCs of each group at passage 16 and passage 30 were plated in 24-well plates and fixed with 4% paraformaldehyde for 10 minutes. The following primary antibodies were used: anti-OCT4 (1 : 100, #sc8628, Santa Cruz), anti-NANOG (1 : 100, #ab21624, Abcam), anti-SOX2 (1 : 500, #ab97959, Abcam), anti-TRA-1-60 (1 : 50, #mab4360, Millipore), anti-TRA-1-81 (1 : 50, #mab4381, Millipore), and SSEA-1 (1 : 50, #mab4301, Millipore). For identifying differentiated embryoid bodies, the following antibodies were used: anti-nestin (#ABD69, Merck), anti-neurofilament (#N41142, Merck), anti-vimentin (#NB500-316, Novus Biologicals), and anti-Gata6 (#ab175349, Abcam).

The cells used for the detection of nuclear markers OCT4, SOX2, NANOG, and GATA6 were first permeabilized with Triton X-100 for 20 minutes and rinsed 3 times with 0.05% Tween 20 in PBS. For blocking OCT4, SOX2, SSEA-1, nestin, neurofilament, vimentin, and GATA6, the cells were treated with 1% bovine serum albumin (#A2153, Sigma-Aldrich) for 1 hour. For blocking NANOG, TRA1-60, and TRA1-81, 10% goat serum (#G9023, Sigma-Aldrich) was added to cells for 30 minutes. The primary antibodies were diluted in the blockage solution and incubated overnight at 4°C. Then, secondary antibodies (1 : 500, #A21044, #A11058 and #A11034, and #A11008, Thermo Fisher) were added and incubated for 1 hour. The cellular nucleus was counterstained with Hoechst (#33342, Thermo Fisher), and the cells were visualized by fluorescence microscopy (EVOS M5000 Imaging System, Life Technologies).

### 2.6. qRT-PCR

Genes related to pluripotency (*OCT4*, *REX-1*, and *NANOG*), metabolism (*GAPDH*, *PFKM*, and *GLUT3*), mitochondrial fusion and fission (*MFN1* and *DNM1L*), low oxygen tension (*HIF1α*, *HIF2α*, and *VEGFA*), and cellular senescence (*TERT* and *CDKN2A*) were evaluated in three colonies of early-passage (EP—P5) and late-passage (LP—P16) cells of each group and in three different cultures of fibroblasts from the same animal cultured in high O_2_ (FH) and low O_2_ (FL). Primers were designed using Primer-BLAST, and the reference sequences were accessed from GenBank (both available online at https://www.ncbi.nlm.nih.gov). The primer sequences are listed in Table 1.

For qRT-PCR, the cells were collected and snap-frozen in liquid nitrogen. The mRNA was extracted with TRIzol (#15596026, Thermo Fisher). Briefly, TRIzol was added to the samples, and after 5 minutes, chloroform was added and retained for 3 minutes. The sample was centrifuged at 15,000 g per 15 minutes at 4°C, and the translucid phase was separated and poured into a new tube. An equal volume of isopropanol was added to the tube, and the sample was maintained at -80°C for 2 hours. The sample was centrifuged, and 1 ml of 75% ethanol was added. After centrifugation, the pellet was allowed to dry, and quantification was performed using a NanoDrop 2000/2000 (Thermo Scientific) spectrophotometer. cDNA synthesis was performed using *a high-capacity reverse transcription kit* (#4368814, Thermo Fisher) according to the manufacturer's instructions.

Relative quantification of the transcript levels was performed using a 7500 Fast Real-Time PCR System with PowerUp SYBR Green Master Mix (#A25777, Thermo Fisher). The reactions were performed at 95°C for 15 minutes and then for 40 cycles at 95°C for 15 seconds, 60°C for 5 seconds, and 72°C for 2 minutes. The melting curve was analyzed to determine the specific amplification of the products, and all reactions were performed in duplicate. The cycle threshold (Ct) values of the target genes were normalized to that of the median Ct value of the HPRT1 and PPIA reference genes, and then, the fold changes were calculated using the 2^(-*Δ*CT)^ equation [26]. Three different eiPSC lineages from each group (H, HL, and L) in early (EP) and late (LP) passages, as well as three fibroblasts cultured in high oxygen tension conditions and three cultured in low oxygen tension conditions, were evaluated. qRT-PCR products were sequenced to assure their specificity, and all presented equine-specific similarity (BLAST analysis).

For statistical analysis, all data were tested to determine whether they were normally distributed using a Shapiro-Wilk test and transformed ([log10(*X*)], [(*X*)^2^], [(*X*)^3^], or [1/(*X*)]) when needed. Then, the data were tested by analysis of variance (ANOVA), and when a significant difference was found, the medians were compared using Tukey's test. The effects were considered significant when *p* < 0.05.

### 2.7. Embryoid Body Assay and Spontaneous Differentiation

For embryoid body formation, eiPSCs in passage 30 (P30) were added to a 6-well plate covered with 0.6% agarose in eiPSC media without bFGF. The formed embryoid bodies were collected for qRT-PCR analysis and plated in IMDM supplemented with 10% fetal bovine serum for 15 days for spontaneous differentiation. The cells were then plated for immunocytochemistry analysis and collected for qRT-PCR.

## 3. Results

### 3.1. Cellular Senescence Analysis

To investigate whether the fibroblasts from the old animal were undergoing a senescence process, the population doubling time (PDT) and qRT-PCR analysis for detecting TERT and CDKN2A, genes encoding a component of the telomerase enzyme and p16 protein, respectively, were performed. The results showed a significantly higher PDT (*p* = 0.0001) in the cells from the old animal (*n* = 1) compared to that from the young animals (*n* = 3), indicating that the young animals' cells proliferated faster than did the old animal's cells. However, it is worth mentioning that the standard deviation of the old animal cell analysis was greater than that of the young animals' (Table 2). Also, no expression of *TERT* was found in the fibroblasts (Table 3), but this gene was expressed in all the eiPSC groups (Table 4), showing telomerase activation in cells after reprogramming. Considering *CDKN2A*, no significant differences were found between groups, and this gene was not expressed in early- or late-passage group L cells, nor in early-passage group HL cells, and it was found to be expressed at lower levels in eiPSCs than in fibroblasts (not significant).

*p* < 0.0001.*N* = number of animals; *R* = number of repetitions; SD = standard deviation; SEM = standard error of the median.

### 3.2. Oxygen Generates Different Profiles of iPSCs

**Figure 1:**
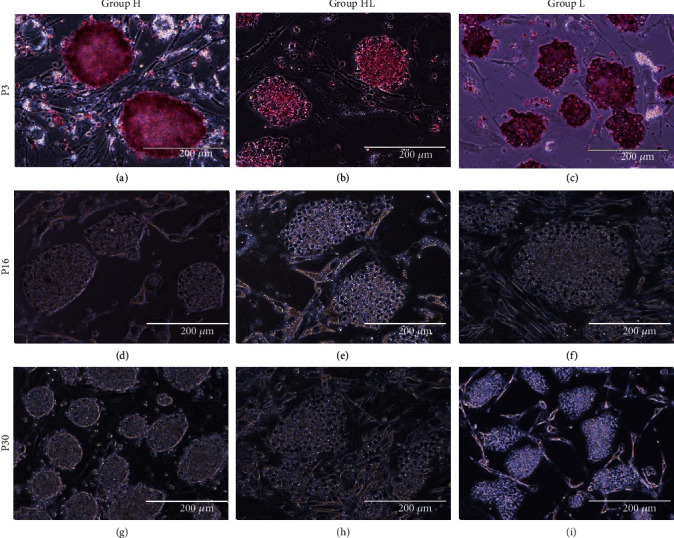
Alkaline phosphatase staining and different colony morphologies in passages 3, 16, and 30: (a, d, g) group H; (b, e, h) group HL; (c, f, i) group L. Scale bars: 200 *μ*m.

After reprogramming, the cells were observed daily, and the first colonies appeared within 16 days. The reprogramming efficiency was higher for the group HL (0.086%), followed by that of group L (0.075%) and of group H (0.059%), showing a higher efficiency for the low oxygen groups than was shown for the high oxygen group. Alkaline phosphatase staining was performed at passage 3, and the colonies of all groups were positively stained (Figure 1). At this time point, differences in the morphology of the colonies of the three groups were observed, with groups HL and L presenting with colonies with a flatter morphology and group H presenting with colonies of a more dome-shaped form with compact cells and well-defined borders (Figure 1, Supplemental Material—Figure 1). These characteristics were also observed in passages 16 and 30 (Figure 1). Cells from the three groups were cultured through passages 36-40 and maintained their typical morphology.

**Figure 2:**
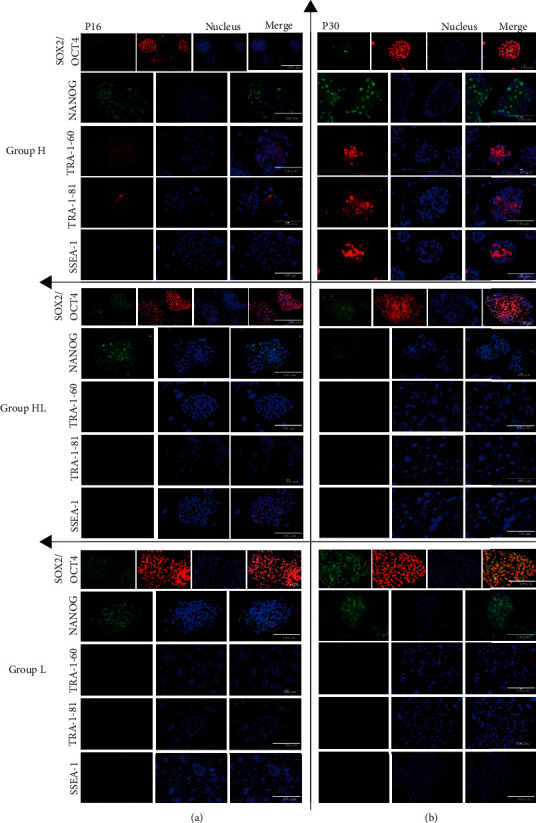
Immunocytochemistry of eiPSC colonies showing positive and negative results for pluripotency markers SOX2, OCT4, NANOG, TRA-1-60, TRA-1-81, and SSEA-1 for groups H, HL, and L in passages 16 (a) and 30 (b). Scale bars: 100 *μ*m, 200 *μ*m, and 400 *μ*m.

Another important difference between groups was evident from the immunofluorescence analysis results. An immunofluorescence test was performed in one colony of each group at passages 16 and 30 (Figure 2), and the results revealed a different pattern of protein expression between colonies cultured at different oxygen tensions. At passage 16, the eiPSC colony from group H had a higher level of pluripotency markers than did that from groups HL and L, being positive for OCT4, NANOG, TRA-1-60, and TRA-1-81 and negative for SOX2 and SSEA-1. The eiPSCs from groups HL and L were positive for OCT4, SOX2, and NANOG and negative for TRA-1-60, TRA-1-81, and SSEA-1. At passage 30, the cells from the colony of group H were positive for OCT4, NANOG, TRA-1-60, TRA-1-81, and SSEA-1 and negative for SOX2. The HL and L groups did not have different protein expression at P30. These results may indicate that group H formed more pluripotent colonies than did group HL or L.

### 3.3. Gene Expression Analysis by qRT-PCR

qRT-PCR analysis was performed in three colonies of each group and three samples in the FH and FL groups, and the results were evaluated in four different ways: comparing fibroblasts (FH×FL) (Table 5), fibroblasts to eiPSCs in early and late passages (fibroblasts×eiPSC EP×eiPSC LP) (Table 3), the effect of oxygen between groups (eiPSC H×eiPSC HL×eiPSC L) (Table 6), and, finally, all groups together (eiPSC EP L×eiPSCs in EP H×eiPSCs EP HL×eiPSC LP L×eiPSC LP H×eiPSC LP HL×FL×FH) (Table 4).

#### 3.3.1. Fibroblast Analysis

The effect of O_2_ tension during *in vitro* culture was first analyzed in fibroblasts to determine a possible environmental effect prior to reprogramming. Interestingly, FL presented a higher variance (dispersion) according to the results of the analysis, except for *CDKN2A*. There was no expression of *REX1*, *NANOG*, *TERT*, or *hOSKM* in the fibroblasts. However, increased expression of *GAPDH* was observed in the FH group (high O_2_) compared to that observed in the FL group (Table 5).

#### 3.3.2. Pluripotency Gene Expression

After reprogramming, all the eiPSCs showed endogenous expression of the pluripotency-related genes. The results from the comparisons of fibroblasts with eiPSCs in the early and late passages (Table 3) show that the *OCT4* (*p* = 0.0030), *REX-1* (*p* < 0.0001), and *NANOG* (*p* = 0053) levels were significantly higher in the eiPSCs than in fibroblasts. The eiPSCs also showed expression of the viral vector (hOSKM, *p* = 0.0008), and the fibroblasts did not express *REX1*, *NANOG*, *TERT*, or *hOSKM*.

#### 3.3.3. Glucose Metabolism

PFKM expression was not different among the eiPSC groups or the eiPSCs compared to the fibroblasts. On the other hand, the eiPSCs had diminished expression of GAPDH (*p* = 0.0002) and GLUT3 (*p* < 0.0001) compared to that in the fibroblasts (Table 3). Furthermore, the analysis of the influence of oxygen in the H, HL, and L groups showed significantly decreased expression of GAPDH (*p* = 0.0491) and GLUT3 (*p* < 0.0034) in group H compared to that of groups L and HL (Table 6).

#### 3.3.4. Mitochondrial Fission and Fusion

*DNM1L* (*DRP1*) was evaluated, but no significant differences were found between groups or between fibroblasts and eiPSCs. On the other hand, eiPSCs presented with diminished expression of *MFN1* (*p* = 0.0004) compared to that expressed in fibroblasts (Table 3), showing a decreased expression after reprogramming.

#### 3.3.5. Low Oxygen Tension Analysis

Regarding low oxygen tension, *HIF1α*, *HIF2α*, and *VEGFA* levels were evaluated. After reprogramming, eiPSCs in early and late passages presented with significantly decreased expression of *HIF1α* (*p* = 0.0004) and *VEGFA* (*p* = 0.0004) compared to their expression in fibroblasts. *HIF2α* was only significantly decreased in early-passage eiPSCs (*p* = 0.0045) compared to the level in fibroblasts (Table 3). Comparing the different oxygen tension groups, no significant difference was observed for *HIF1α*, *HIF2α*, or *VEGFA* expression (Table 6). When comparing all groups (Table 4), no significant difference was observed for HIF1*α*. eiPSCs from early-passage group L did not express *HIF2α*, and in this group, it was possible to observe a significant augmentation of *HIF2α* in late-passage cells compared to the level observed in the early-passage cells. Groups H and HL showed no difference in *HIF2α* expression between the early- and late-passage cells. Compared to fibroblasts, only cells from early-passage groups L and H were significantly decreased in *HIF2α*. VEGFA was significantly decreased after reprogramming, and it was higher in FH than in groups H or HL in the early or late passage; however, this trend was not observed for the cells cultured in low oxygen, as the VEGFA expression in FL was not increased compared to that in group L in either the early or late passage.

### 3.4. Embryoid Body Formation (EB) and Spontaneous Differentiation (SD)

**Figure 3:**
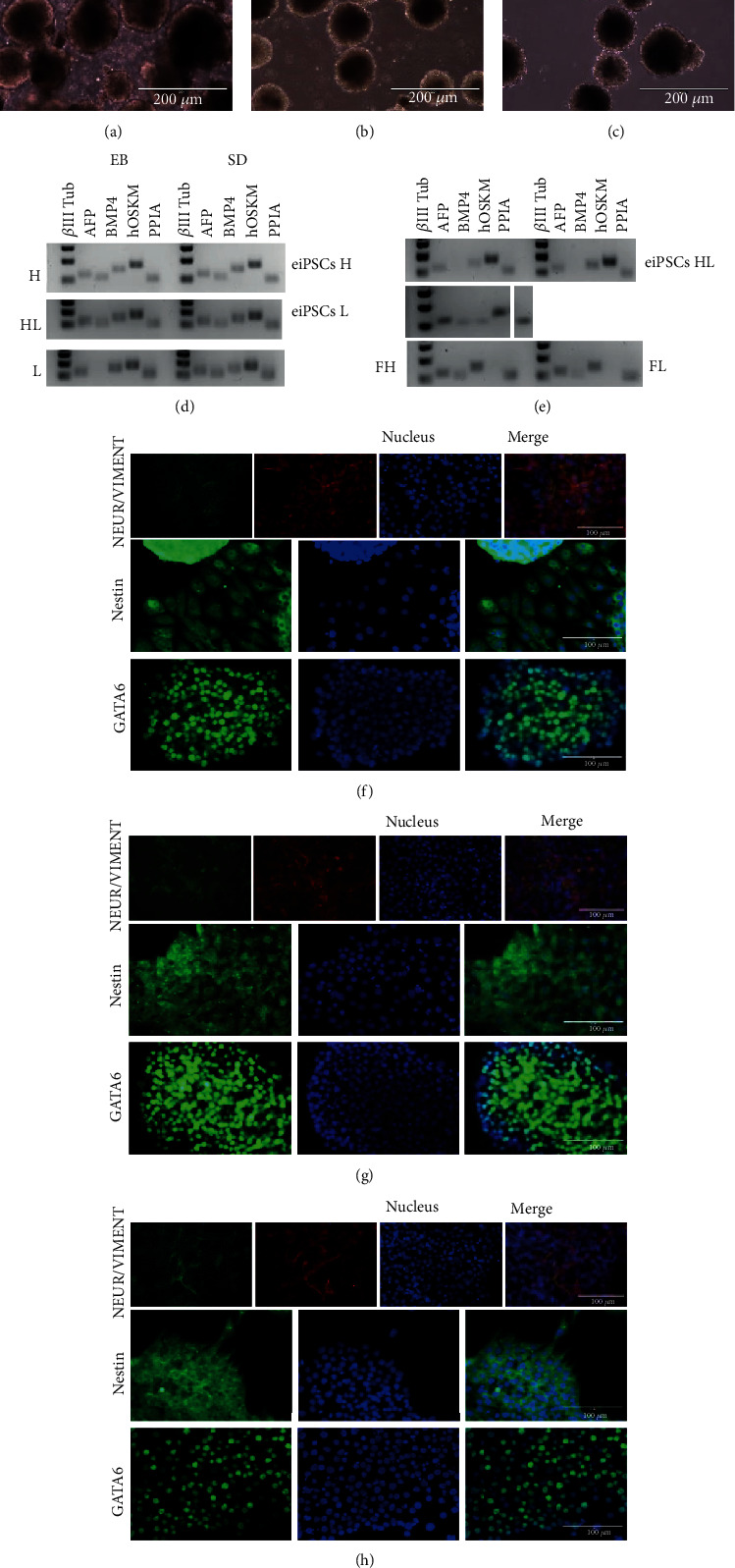
(a–c) Embryoid body (EB) formation from groups H, HL, and L, respectively. (d, e) RT-PCR results for ectoderm gene *β*III-tubulin (*β*III Tub), endoderm gene alpha-fetoprotein (AFP), and mesoderm gene bone morphogenetic protein (BMP4) and for the expression of the lentiviral vector (hOSKM) and housekeeping gene PPIA in embryoid bodies (EB, d, left) and spontaneous differentiation (SD, d, right), from groups H, L, and HL; in eiPSC cells from groups H, HL (e, first row left and right, respectively), and L (second row) and fibroblast (third row) cultured in high (FH, left) and low (FL, right) oxygen. (f–h) Immunocytochemistry of spontaneous differentiation for mesoderm marker vimentin, ectoderm markers nestin and neurofilament, and endoderm marker GATA6, in groups H (f), HL (g), and L (h). Scale bars: 100 *μ*m and 200 *μ*m.

The eiPSCs from groups H, L, and HL were able to form embryoid bodies within 5-7 days of culture (Figures 3(a)–3(c)). Then, the EB were seeded in culture plates to allow spontaneous differentiation (SD). After one week, the spontaneously differentiated cells were plated for immunofluorescence assays. The assay results showed that the cells from groups H, HL, and L were able to differentiate into cells of the ectoderm, mesoderm, and endoderm, as shown by positive staining for ectoderm markers neurofilament and nestin, mesoderm marker vimentin, and the endoderm marker GATA6 (Figures 3(f)–3(h)). Also, the results from the analysis of RT-PCR performed with the cells during EB and SD showed that the three groups expressed *β*III-tubulin, an ectoderm marker, and bone morphogenetic protein 4 (BMP4), a mesoderm marker, during EB and SD. Group L presented the endoderm marker alpha-fetoprotein (AFP) only after SD; however, it was expressed during EB and SD in the other groups (Figure 3(d)).

As a control, we used RT-PCR to also investigate the expression of *β*III-tubulin, AFP, and BMP4 markers in the eiPSCs and the fibroblasts from which the EB were derived (Figure 3(e)). The fibroblasts showed the expression of all three markers. eiPSCs were positive for *β*III-tubulin and BMP4 in all groups and for AFP only in group L. The viral vector was still expressed even after embryoid body differentiation.

## 4. Discussion

### 4.1. Cellular Senescence Analysis

Previous studies have shown that telomere shortening occurs with aging *in vivo* and *in vitro*, and this process is related to decreased cellular proliferation and increased senescence [11, 27, 28]. Additionally, iPSCs present with more extensive telomere elongation than do somatic cells [29]. These previous data are in accordance with the telomerase activation observed in the eiPSCs in this study, and although the size of the telomeres was not measured, the lower proliferation rate of cells from the old animal may reflect a lack of telomerase.

As senescence leads to the upregulation of the cyclin-dependent kinase inhibitors p21 and p16 [30] and because *CDKN2A* is the gene encoding p16, this gene was also evaluated in the assessment of cellular senescence. Although no significant differences were found between groups for this gene, *CDKN2A* was not expressed in early- or late-passage group L cells nor in early-passage group HL cells, and it was found to be expressed at lower levels in eiPSCs than it was in fibroblasts. p16 is involved in cell cycle arrest [31] and is not highly expressed in cells with an active cell cycle. However, as the quantity of *CDKN2A* was extremely low in most of our cell samples, the standard error was remarkably high compared to the expression level of this gene. Thus, a more accurate technique than qRT-PCR, such as an assessment of protein levels, should be employed to test the expression of this gene in these cells. However, any result associated with a lack of *TERT* expression and/or a low fibroblast proliferation rate would indicate that these cells are heading to a senescent state, and reprogramming them to pluripotency might reverse this direction.

Considering the PDT under high and low oxygen tension conditions, there were no significant differences between the FH and FL groups (*p* = 0.957). It is important to consider that hypoxia could also induce cytotoxicity, and the response to hypoxia varies between different types of cells. It was already described that 1% oxygen cultivation inhibits the proliferation of human dermal fibroblasts and could even lead to cell death, while the same 1% O_2_ had little effect on the mouse embryonic stem cell (MEF) proliferation [16]. In equine species, a previous study in dermal fibroblasts showed significantly increased proliferation of cells cultured in 1% O_2_ compared to those cultured in atmospheric oxygen (20% O_2_) [32]; however, in the present work, we could not observe effects of low oxygen in the proliferation of the equine fibroblasts cultured in 5% or 20% oxygen, as shown by our population doubling time results.

No previous studies were found regarding horse cell proliferation during aging. In humans, controversial results were published in literature, with some reports showing an influence of aging on cellular proliferation [33, 34] and others showing no effect [35, 36].

### 4.2. Oxygen Generates Different Profiles of iPSCs

Previous studies reported a higher reprogramming efficiency of human and mouse cells in 5% O_2_ culture conditions compared to those in 20% O_2_ [16, 37]. However, it is important to consider more characteristics of the obtained colonies than the reprogramming efficiency because, as we show in the current study, a higher efficiency does not necessarily correlate with a more pluripotent colony.

Regarding the differences in morphology of cells cultured in different oxygen tensions, no previous results were found in equine species, but dissimilar results were reported for hESCs [18, 38, 39], which had a more compact morphology and well-defined colony borders in 5% O_2_ than did hESCs cultured in 20% O_2_.

The OCT4 and SOX2 antibodies used in this study were not equine-specific; therefore, there might have been some expressed exogenous human OCT4 and SOX2 proteins that were positively stained in the immunofluorescence test. However, the membrane proteins TRA-1-60, TRA-1-81, and SSEA-1 and the nuclear protein NANOG were not present in the viral vector used, and therefore, their expression was equine-specific. The finding that the colony of group H cells expressed OCT4, but not SOX2, may indicate that even though the viral vector was probably producing its proteins (OCT4, SOX2, KLF4, and c-Myc, once the vector expression was active), probably the SOX2 protein was not produced by these cells or was produced in a quantity sufficiently low that it was not detectable. Indeed, it is known that species-specific differences are present for pluripotency acquaintance and maintenance; however, the requirements for specific signaling pathways and interactions between transcription factors are not completely unraveled yet [40, 41]. Interestingly, in contrast to our results, a previous study [42] found no differences in POU5F1 (OCT4), SOX2, TRA-1-60, and TRA-1-81 in the 5% and 20% cultures.

Combining the results of the cellular morphology and immunofluorescence assays, it is possible to conclude that the low oxygen environment did not enhance the pluripotency of our eiPSCs, as the cells cultured in high oxygen presented some naïve characteristics, while the cells cultured in low oxygen presented with a more primed profile [42]. A previous study showed significant differences between the naïve state of mouse embryonic stem cells (mESCs) and primed mouse epiblast stem cells (EpiSCs) and primed human ESCs (hESCs) [40]. The authors showed that the primed cells preferentially activated glycolytic metabolism and had diminished mitochondrial function compared to the naïve cells. On the other hand, an augmentation of oxidative phosphorylation (OXPHOS) in naïve cells, compared to that in primed cells, was previously reported [41]; these authors showed that, although both naïve and primed pluripotent cells preferentially depend on glycolysis metabolism to produce energy, naïve cells also undergo OXPHOS and consequently show less glycolytic activity than is shown by the primed cells [20]. These studies show a close regulation of metabolism in primed and naïve cells and highlight the importance of additional studies in the field, primarily studies among different species.

### 4.3. Gene Expression Analysis by qRT-PCR

#### 4.3.1. Fibroblast Analysis

It is important to consider that, in addition to the role of GAPDH in glycolysis, several other equally important functions are attributed to it, as reviewed in previous articles [43–45]. GAPDH is inhibited by oxidative stress [46], which can lead to cellular aging and apoptosis [45]. In the current study, a higher expression of this gene in the fibroblasts cultured in high O_2_ compared to that of the fibroblasts cultured in low O_2_ was verified, which could indicate that even the fibroblasts in high O_2_ conditions do not undergo oxidative stress, possibly because of compensatory mechanisms activated by the fibroblasts. However, this gene may be regulated in multiple ways in fibroblasts and during reprogramming to pluripotency.

#### 4.3.2. Pluripotency Genes

Some previous works reported increased gene expression of pluripotency-related genes in mouse and human iPSCs and ESCs cultured in a low oxygen atmosphere [16, 39]. These cited studies found diminished expression of *POU5F1* (*OCT4*), *SOX2*, and *NANOG* mRNA in cells cultured in 20% oxygen compared to that in cells cultured in 5% O_2_. Our results, in contrast, showed no significant differences in *NANOG* expression in the 5%, 20%, and 20% to 5% groups, and for OCT4, a decrease in expression in late-passage cells from group H compared to that in the late-passage cells from group HL was observed (Table 4). This same pattern of expression was observed in the hOSKM viral vector, which might indicate that the endogenous expression of OCT4 may be regulated by the exogenous OCT4 source.

Interestingly, REX-1 showed a passage effect, with a higher expression in late-passage cells than in early-passage cells from the H and L groups. REX1 is a transcription factor with important roles in maintaining the typical iPSC morphology in human cells, inducing an upregulation of pluripotent markers and a downregulation of differentiation markers, influencing EB and teratoma formation potential, with a marked influence in mesoderm lineages, and it also has a role in metabolic cellular processes, as its deletion causes increased oxygen consumption, indicating higher oxidative phosphorylation (OXPHOS) activity, downregulation of glycolytic genes, and decreased lactate production, indicating reduced glycolysis [47]. Thus, the augmentation of this gene found in the late-passage cells from the H and L groups compared to its expression in the early-passage cells in the current work may indicate enhanced reprogramming of the eiPSCs over time.

#### 4.3.3. Glucose Metabolism

As regulation of the glycolytic pathway is considered to be of high importance for iPSC formation [48], PFKM, a phosphofructokinase critical for the second key step of glycolysis [49]; GAPDH, a gene acting in the sixth step of the glycolysis process [45]; and GLUT3, the gene encoding a membrane protein crucial for glucose transport to the cell [50], were evaluated in the current work.

The decreased expression of GAPDH and GLUT3 in eiPSC compared to that in the fibroblasts was not expected, as there is a shift from oxidative phosphorylation to glycolysis for energy production during the reprogramming into the pluripotent cells [51, 52], and with a higher energetic demand for ATP by proliferating cells, the glycolytic pathway was thought to be more active and glucose transport to the cell was expected to increase. It is possible that genes other than those evaluated are involved in glucose uptake and metabolism.

On the other hand, a decreased expression of GAPDH and GLUT3 was observed in group H compared to groups HL and L. These results suggest that, after reprogramming, the cells cultured in high oxygen use the glycolytic pathway less than those cultured under low oxygen conditions. This decrease in GLUT3 expression in cells in 5% O_2_ compared to that in cells in 20% O_2_ was previously reported in human embryonic stem cells (hESCs) [50]. Additionally, silencing GLUT3 in hESCs led to a decrease in glucose uptake and lactate production, along with a correlated decrease in OCT4 [50], linking glucose transport to pluripotency. In the current study, a decrease in GLUT3 expression was observed after reprogramming, but an augmentation of this gene in the cells cultured in low oxygen conditions was observed. Together, these results suggest modulation of cellular glycolysis metabolism, with an augmentation of glucose uptake and glycolysis under low oxygen tension.

Interestingly, as there was no significant difference in the expression of PFKM, a gene responsible for the initial steps of glycolysis, but there was a significant decrease in GAPDH in group H compared to that in groups L and HL, it is possible that GAPDH is regulating other important functions in addition to being involved in the glycolysis pathway. Unfortunately, GAPDH was considered a housekeeping gene for a long time, and no studies demonstrating its role in iPSC cells are available.

#### 4.3.4. Mitochondrial Fission and Fusion

Mitochondrial fusion and fission are two important processes that guarantee the number and shape of these organelles in cells, as fission is the process of the division of mitochondria, and fusion is the opposite operation. The equilibrium between these two processes regulates the overall morphology and consequently the function of mitochondria in cells [53, 54]. The fission process is essential for reprogramming [55], and a previous study has shown an increase in fission gene *DNM1* expression and a decrease in the fusion *MFN1/2* and *CHCHD3* gene expression during reprogramming [56]. These authors also demonstrated that *DNM1L* (*DRP1*), *FIS1*, and *MFF* fission genes remained unchanged after reprogramming. In accordance with these findings, in the current work, *DNM1L* (*DRP1*) was evaluated, but no significant differences were found between groups or between fibroblasts and eiPSCs. However, other important fission genes, such as *DNM1*, *MiD51*, *MFF*, and *GDAP1*, were not evaluated in the current work.

*MFN1*, in its turn, was diminished in eiPSC compared to the fibroblasts, showing, as expected, reduced expression during reprogramming. Previous work has shown that the suppression of *MFN1/2* has an influence on metabolic transitions with the downregulation of OXPHOS genes and the upregulation of glycolysis genes and the augmentation of glycolysis metabolites, with a consequent impact on reprogramming efficiency [56].

#### 4.3.5. Low Oxygen Tension Analysis

*HIF1α* and *HIF2α* are important genes for reprogramming, as they are upregulated during this process in both high and low oxygen conditions. Reprogramming is impaired by *HIF2α* knockdown, as the lack of this gene negatively influences the metabolic shift, but the prolonged stabilization of *HIF2α* also impairs reprogramming [37]. These results show that in addition to the expected role of *HIF* in controlling hypoxia-related genes, this transcription factor plays an equally important role in the reprogramming process.

A previous study showed that the long-term response to low oxygen tension was regulated by *HIF2α* but not *HIF1α* [39], showing that these genes were not necessarily expressed in cells with low oxygen tension. This might explain why no differences were found for *HIF1α* and *HIF2α* under the different oxygen tension conditions, as the cell possibly was metabolically stable after being in the respective oxygen conditions for a long time, and as significant differences were found for these genes after reprogramming, their main role was in reprogramming to pluripotency rather than in regulating the cells under the 5% O_2_ condition. Another possible explanation suggests that these factors have a complex regulatory action that is dependent on several proteins, such as prolyl hydroxylases [57], which cause their degradation in less than 5 minutes in 21% O_2_ [58]; therefore, our mRNA expression analysis may not be sufficient to detect the shifts in the expression of these genes, and methods other than the mRNA expression evaluation are necessary to further analyze their roles in the reprogramming and low oxygen culturing of eiPSCs.

As *HIF* genes are related to the metabolic shift that occurs in reprogramming, it was expected that they would influence cells in the naïve and primed states, as it is known that these different states are metabolically different [20]. Indeed, a previous study has demonstrated this influence, as there was an upregulation in naïve cell markers and downregulation in primed cell markers in *HIF1α*-knockout hESCs [17]. These data suggest that a hypoxic environment favors a primed state of iPSCs. Additionally, another study showed that *HIF1α* induced metabolic and morphological changes in mESCs, rendering them in a primed state, and it was increased in the primed cells causing the higher glycolysis metabolism in these cells than in the naïve cells [40]. Although there was no significant difference in *HIF1α* in cells under different oxygen treatments in the current work, we found different morphologies and immunofluorescence markers in the colonies of the different groups, as the cells cultured in low oxygen presented with a more primed morphology. This finding suggests that, even though we did not observe a difference in HIF1*α* expression, posttranscriptional regulation may have caused more primed features to be expressed in the colonies of groups HL and L.

### 4.4. Embryoid Body Formation (EB) and Spontaneous Differentiation (SD)

The fact that eiPSCs were positive for some differentiation markers shows an incomplete silencing of the differentiation-related genes after reprogramming. Only group L cells expressed the endoderm marker AFP, suggesting that culturing eiPSCs in low oxygen for long periods may influence the capacity of these cells to silence the endoderm gene. In accordance with this finding, a previous report indicated that culturing cells in lower oxygen tension levels favors differentiation in the endoderm lineage [59].

This result reinforces the supposition that the alteration of the oxygen atmosphere leads to different iPSC profiles, and this preference could be leveraged to favor guided differentiation of cells prior to their possible clinical application or for use in studies *in vitro*. Additionally, in a study of pluripotent stem cells cultured in different oxygen atmospheres, it is important to determine the criteria that define a low and high oxygen culture for each kind of cell, noting evident differences among species.

## 5. Conclusion

The current study successfully reprogrammed the pluripotency of fibroblasts from an animal more than 20 years old. The generated eiPSCs were positive for alkaline phosphatase and, according to the immunocytochemistry evidence, for OCT4 and NANOG (all groups), SOX2 (groups HL and L), and TRA-1-60, TRA-1-81, and SSEA-1 (only group H). Additionally, the cells from all groups showed endogenous expression of the pluripotency-related genes *OCT4*, *REX1*, and *NANOG* and underwent EB and spontaneous differentiation, to generate cells of origin for the three embryonic germ layers: endoderm, ectoderm, and mesoderm. Moreover, the population doubling time and the results from the TERT and *CDKN2A* expression analyses suggest that, after reprogramming, the eiPSCs reverted to a senescent state as fibroblasts.

Important differences were observed between the eiPSCs formed under the different oxygen tension conditions. The results from the morphology, immunofluorescence, and gene expression assays suggest that different eiPSC profiles were acquired in each environment. These results are unprecedented for equine and show that the low oxygen tension culture does not seem to favor pluripotency for cells from these species. However, it is important to highlight that oxygen tension had an influence on forming the different profiles of the eiPSCs.

## Figures and Tables

**Table 1:** Primer sequences used for the detection of pluripotency, senescence, embryoid body evaluation, and genes involved in metabolism, lower oxygen tension, and mitochondrial remodeling.

Target gene	Primer forward	Primer reverse	Product size	NCBI reference
OCT4	AGAAGGACGTGGTACGAGTG	GTGCCAGGGGAAAGGATACC	138	XM_001490108.6
REX-1	TGGAGGAATATCCAGCGTTGA	GCTTTCCCACATTCTGCACATA	213	XM_001489519.4
NANOG	CTCGATTTGGGCAGTGGCTA	CGAGCCCTCTAGAATCCGTC	117	XM_023643093.1
DNM1L	AGATGTGCCGGTTCCTGTAG	CTCTCTGTTCACGGGCAGAC	50	XM_023643346.1
MFN1	GGCAAAATGTGCCCTCTTGA	TCTGTACCCGGACTGTCCAC	57	XM_023623336.1
HIF1*α*	CACCAGAGCCTAACAGTCCC	AGTCCGTGTCCTGAGTGGAA	141	XM_023627857.1
HIF2*α*	GTTGTCAGGCATGGCAAGTC	TCAATTCGGGCAGCAGGTAA	65	XM_005600005.3
VEGFA	TGCGGATCAAACCTCACCAA	GCCCACAGGGATTTTCTTGC	114	NM_001081821.1
PFKM	GCTCAGTGAGACAAGGACCC	TTCCTGTCAATCGCACCCTC	72	NM_001081922.2
GLUT3	CCGTCCCACTGAGGTGTTAC	ATTGAATTGCGCCTGCCAAA	126	XM_023643091.1
GAPDH	GTTTGTGATGGGCGTGAACC	ATCGCGCCACATCTTCCC	205	NM_001163856.1
TERT	CTGAGGGACGCTGTTGTCAT	ACTGGACATAGGACTTGCCG	127	XM_023625881.1
CDKN2A	GGAGGTTGCCCGGTATCTG	ATCACTTAACGTCTGCGGGG	127	XM_023627432.1
*β*III-Tubulin	AGCCATGTAGCCACTGACAC	TGGGTAGACTCACATCGGGT	123	XM_023637577.1
*α*-Fetoprotein	AGCAAGTGGCTGGCCTTATT	CATGGCCTCCTGTTGGCATA	112	NM_001081952.1
BMP4	CGCTTCTTTCCCTGATGGGAT	ATGGCTCCATAGGTCCCTGC	150	NM_001163970.1
PPIA	ATGTGTCAGGGTGGTGACTTC	GGACCGGTGTGTTTCAGGAT	104	XM_001496943.5
HPRT1	GCTTCCTCTTCCTCAGACGG	TCACTAATCACGACACTGGGG	80	XM_023634464.1

**Table 2:** Effect of age in population doubling time (PDT).

	*N*	*R*	Mean	SD	SEM	
Young	3	25	24.0598682	4.9050917	0.9810183	B
Old	1	10	45.3293396	26.3544309	8.3340028	A

**Table 3:** Gene expression analysis comparing fibroblasts with eiPSCs in the early passage (EP eiPSCs—passage 5) and late passage (LP eiPSCs—passage 16).

Gene function	Gene	Fibroblasts	EP eiPSCs	LP eiPSCs
Pluripotency	OCT4	0.0058 ± 0.0022^B^	0.5177 ± 0.1428^A^	0.4808 ± 0.1357^A^
	REX1	3.5300 × 10^−5^ ± 6.0880 × 10^−5^^C^	0.0312 ± 0.0092^B^	0.0415 ± 0.0033^A^
	NANOG	2.6235 × 10^−5^ ± 3.4150 × 10^−5^^B^	0.6406 ± 0.2650^A^	0.4128 ± 0.2800^A^
Viral vector	hOSKM	5.7930 × 10^−6^ ± 1.3059 × 10^−5^^B^	0.6790 ± 0.2864^A^	0.5837 ± 0.2929^A^
Glucose metabolism	GAPDH	4.4190 ± 1.2198^A^	1.0521 ± 0.1494^B^	1.1898 ± 0.6966^B^
	GLUT3	0.4220 ± 0.2182^A^	0.1768 ± 0.0543^B^	0.1478 ± 0.0548^B^
	PFKM	0.0674 ± 0.0534	0.0636 ± 0.0221	0.0686 ± 0.0162
Mitochondrial fusion and fission	MFN1	0.0837 ± 0.0697^A^	0.0191 ± 0.0059^B^	0.0232 ± 0.0100^B^
	DNM1L	0.0274 ± 0.0164	0.0165 ± 0.0095	0.0192 ± 0.0155
Lower O_2_ tension	HIF1A	2.0370 ± 1.5210^A^	0.1366 ± 0.0122^B^	0.1047 ± 0.0665^B^
	HIF2A	0.1057 ± 0.0768^A^	0.0043 ± 0.0092^B^	0.0047 ± 0.0038^AB^
	VEGFA	0.1432 ± 0.0735^A^	0.0298 ± 0.0203^B^	0.0337 ± 0.0243^B^
Cellular senescence	TERT	9.8464 × 10^−6^ ± 2.4119 × 10^−5^^B^	0.0077 ± 0.0049^A^	0.0076 ± 0.0043^A^
	CDKN2A	0.0106 ± 0.0157	0.0013 ± 0.0017	8.4083 × 10^−4^ ± 0.0011

^A-C^Superscript capital letters represent differences (*p* < 0.05) between columns on the same row.

**Table 4:** Gene expression analysis comparing eiPSCs in early passage (EP) and late passage (LP) from the H (20% O_2_), HL (20% to 5% O_2_), and L (5% O_2_) groups and fibroblasts in 20% O_2_ (FH) and 5% O_2_ (FL).

Gene function	Gene	eiPSCs EP L	eiPSCs EP H	eiPSCs EP HL	eiPSCs LP L	eiPSCs LP H	eiPSCs LP HL	FL	FH
Pluripotency genes	OCT4	0.5716 ± 0.1485^AB^	0.5175 ± 0.1913^AB^	0.4640 ± 0.1193^ABC^	0.4454 ± 0.0931^ABC^	0.3802 ± 0.1265^BCD^	0.6167 ± 0.0652^A^	0.0057 ± 0.0023^CD^	0.0058 ± 0.0027^D^
	REX1	0.0280 ± 0.0086^BC^	0.0294 ± 0.0108^BC^	0.0363 ± 0.0094^AB^	0.0425 ± 0.0039^A^	0.0392 ± 0.0007^AB^	0.0436 ± 0.0040^A^	4.9401 × 10^−5^ ± 8.5565 × 10^−5^^C^	2.1200 × 10^−5^ ± 3.6720 × 10^−5^^C^
	NANOG	0.7612 ± 0.3182^A^	0.6178 ± 0.3671^A^	0.5427 ± 0.0889^A^	0.3186 ± 0.2646^AB^	0.2567 ± 0.2050^AB^	0.6630 ± 0.2402^A^	2.1790 × 10^−5^ ± 3.7741 × 10^−5^^B^	3.0680 × 10^−5^ ± 3.7841 × 10^−5^^B^
Viral vector	hOSKM	0.7126 ± 0.3740^AB^	0.6121 ± 0.2926^AB^	0.7122 ± 0.3040^AB^	0.5371 ± 0.1428^AB^	0.3443 ± 0.1358^BC^	0.8698 ± 0.3039^A^	7.9334 × 10^−7^ ± 1.3741 × 10^−6^^C^	1.0793 × 10^−5^ ± 1.8694 × 10^−5^^C^
Glucose metabolism	GAPDH	1.0985 ± 0.0623^B^	0.9256 ± 0.1607^BC^	1.1321 ± 0.1507^B^	1.5894 ± 1.1018^B^	0.8230 ± 0.1389^C^	1.1407 ± 0.2066^B^	3.5014 ± 0.3737^A^	5.3366 ± 1.0268^A^
	GLUT3	0.1965 ± 0.0494^BC^	0.1290 ± 0.0258^C^	0.2049 ± 0.0590^BC^	0.1635 ± 0.0421^BC^	0.0913 ± 0.0338^C^	0.1939 ± 0.0164^BC^	0.3971 ± 0.3196^AB^	0.4469 ± 0.1227^A^
	PFKM	0.0638 ± 0.0059	0.0823 ± 0.0238	0.0597 ± 0.0036	0.0463 ± 0.0207	0.0738 ± 0.0111	0.0709 ± 0.0270	0.0728 ± 0.0750	0.0621 ± 0.0376
Mitochondrial fusion and fission	MFN1	0.0160 ± 0.0013	0.0226 ± 0.0099	0.0185 ± 0.0022	0.0283 ± 0.0164	0.0206 ± 0.0004	0.0196 ± 0.0064	0.0638 ± 0.0765	0.1037 ± 0.0714
	DNM1L	0.0286 ± 0.0270	0.0143 ± 0.0047	0.0146 ± 0.0029	0.0149 ± 0.0074	0.0130 ± 0.0026	0.0215 ± 0.0156	0.0265 ± 0.0223	0.0281 ± 0.0170
Lower O_2_ tension	HIF1A	0.1450 ± 0.0040	0.1250 ± 0.0055	0.1396 ± 0.0150	0.0604 ± 0.0145	0.1347 ± 0.0703	0.1191 ± 0.0890	1.5153 ± 1.5758	2.5586 ± 1.5762
	HIF2A	9.1975 × 10^−4^ ± 2.3422 × 10^−4^^C^	0.0013 ± 0.0010^BC^	0.0106 ± 0.0157^A^	0.0067 ± 0.0044^A^	0.0047 ± 0.0049^AB^	0.0029 ± 0.0016^A^	0.1053 ± 0.1126^A^	0.1062 ± 0.0453^A^
	VEGFA	0.0294 ± 0.0163^BC^	0.0165 ± 0.0165^C^	0.0435 ± 0.0238^BC^	0.0551 ± 0.0286^ABC^	0.0137 ± 0.0034^C^	0.0322 ± 0.0156^BC^	0.1057 ± 0.0935^AB^	0.1807 ± 0.0238^A^
Cellular senescence	TERT	0.0058 ± 0.0021^AB^	0.0085 ± 0.0092^A^	0.0088 ± 0.0011^A^	0.0093 ± 0.0045^A^	0.0037 ± 0.0022^AB^	0.0097 ± 0.0039^A^	1.9693 × 10^−5^ ± 3.4109 × 10^−5^^B^	0.0000 ± 0.0000^B^
	CDKN2A	7.7639 × 10^−4^ ± 0.0012	0.0014 ± 0.0016	3.9345 × 10^−4^ ± 3.6537 × 10^−4^	4.3806 × 10^−4^ ± 5.5601 × 10^−4^	0.0023 ± 0.0028	0.0010 ± 0.0011	0.0140 ± 0.0213	0.0072 ± 0.0114

^A-C^Superscript capital letters represent differences (*p* < 0.05) between columns on the same row.

**Table 5:** Gene expression analysis comparing fibroblasts cultured in 20% O_2_ (FH) to those cultured in 5% O_2_ (FL) prior to reprogramming.

Gene function	Gene	FH	FL
Pluripotency	OCT4	0.0058 ± 0.0026	0.0040 ± 0.0034
Glucose metabolism	GAPDH	5.3366 ± 1.0268^A^	3.5014 ± 0.3737^B^
	GLUT3	0.4469 ± 0.1227	0.3971 ± 0.3196
	PFKM	0.0621 ± 0.0376	0.0728 ± 0.0750
Mitochondrial fusion and fission	MFN1	0.1037 ± 0.0714	0.0638 ± 0.0765
	DNM1L	0.0281 ± 0.0170	0.0348 ± 0.0213
Lower O_2_ tension	HIF1A	2.5586 ± 1.5762	1.5153 ± 1.5758
	HIF2A	0.1062 ± 0.0453	0.1053 ± 0.1126
	VEGFA	0.1807 ± 0.0238	0.1057 ± 0.0935
Cellular senescence	CDKN2A	0.0072 ± 0.0114	0.0140 ± 0.0213

^A,B^Superscript capital letters represent the differences (*p* < 0.05) between columns on the same row.

**Table 6:** Gene expression analysis comparing the three oxygen treatments, eiPSCs H (20% O_2_), eiPSCs HL (20% to 5% oxygen), and eiPSCs L (5% O_2_).

Gene function	Gene	eiPSCs H	eiPSCs HL	eiPSCs L
Pluripotency genes	OCT4	0.4489 ± 0.1634	0.5403 ± 0.1200	0.5085 ± 0.1306
	REX1	0.0343 ± 0.0087	0.0392 ± 0.0080	0.0353 ± 0.0099
	NANOG	0.4372 ± 0.3314	0.6029 ± 0.1749	0.5399 ± 0.3568
Viral vector	hOSKM	0.4782 ± 0.2513	0.7910 ± 0.2852	0.6249 ± 0.2709
Glucose metabolism	GAPDH	0.8743 ± 0.1456^B^	1.1355 ± 0.1485^A^	1.3439 ± 0.7480^A^
	GLUT3	0.1101 ± 0.0339^B^	0.1994 ± 0.0392^A^	0.1833 ± 0.0446^A^
	PFKM	0.0780 ± 0.0172	0.0653 ± 0.0183	0.0550 ± 0.0166
Mitochondrial fusion and fission	MFN1	0.0216 ± 0.0064	0.0189 ± 0.0036	0.0222 ± 0.0124
	DNM1L	0.0137 ± 0.0035	0.0180 ± 0.0107	0.0218 ± 0.0192
Lower O_2_ tension	HIF1A	0.1299 ± 0.0449	0.1294 ± 0.0582	0.1027 ± 0.0473
	HIF2A	0.0030 ± 0.0037	0.0068 ± 0.0109	0.0038 ± 0.0042
	VEGFA	0.0151 ± 0.0107	0.0379 ± 0.0190	0.0422 ± 0.0251
Cellular senescence	TERT	0.0061 ± 0.0065	0.0093 ± 0.0026	0.0076 ± 0.0037
	CDKN2A	0.0018 ± 0.0021	8.2483 × 10^−4^ ± 3.3674 × 10^−4^	6.0723 × 10^−4^ ± 8.8168 × 10^−4^

^A,B^Superscript capital letters represent differences (*p* < 0.05) between columns on the same row.

## Data Availability

The data used to support the findings of this study are included within the article.

## Supplementary Materials

Supplementary Materials Supplemental Material—Figure 1: amplification of the borders of the colonies: colonies from group H have more defined borders and dome-shaped morphology, visually assessed, than groups HL and L.
