# Supplementary material for: In Vitro Induction of Pluripotency from Equine Fibroblasts in 20% or 5% Oxygen
Source: Stem Cells Int. 2020 Nov 26;2020:8814989. doi: 10.1155/2020/8814989 (PMC7785345; doi:10.1155/2020/8814989)
Supplement: Supplementary Materials — Supplemental Material—Figure 1: amplification of the borders of the colonies: colonies from group H have more defined borders and dome-shaped morphology, visually assessed, than groups HL and L. [file 8814989.f1.docx]

*
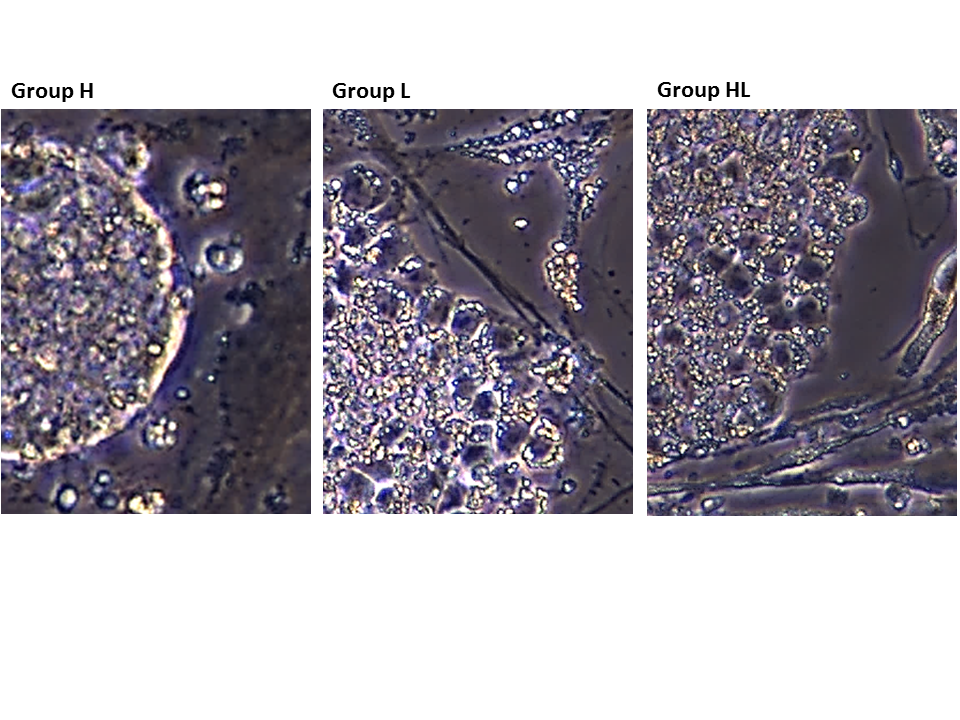
*

**Supplemental Material – Figure 1**

Amplification of the borders of the colonies: colonies from group H have more defined borders and dome-shaped morphology, visually assessed, than groups HL and L.
